# Annotations

**Published:** 1886-11-06

**Authors:** 


### ANNOTATIONS.

Residential Accommodation for Medical Students.

A GOOD deal of correspondence has recently appeared in the public press with reference to the Control and Housing of Medical Students, and the propriety of establishing colleges in connection with the various clinical hospitals. It is pretty well known that an approach to the college system has been partially carried out at several of the Metropolitan medical schools, but not to the extent of giving it a fair trial, and there are grave doubts whether the scheme as a whole is likely to succeed in London. The expenses involved by a collegiate residence within the walls, or in the neighbourhood of the hospital, computing them at the lowest average, would probably exceed the charges for board and lodging outside, and the numbers able to avail themselves of the privilege must necessarily be limited. On the other hand, it is a manifest advantage for the student to sleep away from the bustle and toil of London; and nearly every hospital is now within a stone's-throw of a railway station in immediate and frequent communication with a more or less distant suburb, where lodgings can be had both good and cheap, and where there is little temptation to be drawn into the maelstrom of London profligacy. We are far from participating in the too readily received opinion that medical students as a class are worse conducted than young men of a similar age among other sections of the community. But experience has shown that when banded together in masses, their natural exuberance of animal spirits is liable to outrun their discretion, and to render them sometimes obnoxious to their neighbours. The residential college system, if properly disciplined, would probably prevent such occurrences, so far as those in residence are concerned; at the same time it is more than doubtful whether the congregating together of a large number of young men in the heart of a populous locality, with free licence to invade the hospital at all hours of the day or night, can be regarded other than as a dangerous innovation. The system pursued in some hospitals (not in many), where a large number of senior students are permitted to board and lodge in the building on the strength of holding appointments of some kind, with liberty to entertain their friends and fellow students, has more than once given rise to serious scandal. It would conduce much to the general advantage, if all such gatherings, which lead to turbulence and disorder, were suppressed by a strong hand. We are induced to make these remarks, not less in the interests of the students themselves, than of the hospitals and schools to which they are attached, the reputation and welfare of which depend materially on the position they occupy in the eyes of the public as regards good order and government.

Infected Cabs.

With the view of assisting the Metropolitan Police to check the spread of infectious diseases, the Committee of the Seamen's Hospital have made arrangements to communicate to the Commissioners the number of each cab which brings a patient to the hospital suffering from any infectious or contagious disease. This will ensure that every cab so used shall be immediately and properly disinfected. How often it happens that a member of a family is struck down suddenly by some disease of this kind, and that the source of infection cannot be detected. The Metropolitan Asylums Board have established a complete system of ambulances for the conveyance of patients so infected, but this is not enough to prevent the use of street cabs. It therefore becomes necessary to take other precautions, and the system initiated by the Seamen's Hospital Committee should be adopted at every hospital. In this way many cases of mysterious infection would be prevented, and the public health must be thereby promoted. The thanks of the whole community are due to the Seamen's Hospital for this public-spirited act, which will no doubt commend itself to the common sense of the managers of all institutions.

**Lodging-house Keepers and their Consciences.** The case of Professor Nettleship, which will be familiar to the readers of the daily papers, serves to emphasise a danger to which all annual holiday seekers are peculiarly liable. Mr. Nettleship desired to obtain lodgings at New Quay, and was strongly recommended by two highly respectable lodging-house keepers there to take a portion of a certain farmhouse. The farmer's child was at the very time suffering from scarlet fever, and the process of "peeling" was in full operation when Mr. Nettleship, with his family, took possession of the apartments. One of the professor's own children was speedily attacked by the fever, and the family holiday turned into a period of great suffering for the child and of most painful anxiety for the parents. Of course, all children are liable to come into contact with persons suffering from infectious diseases, but the grievous part of Professor Nettleship's case was, that the farmer knew quite well that his child was in the infectious stage of scarlet fever, and both the "highly respectable lodging-house keepers" knew it also. Persons of strong parental feelings will naturally say that such conduct is shameful, and ought to be severely punished. No doubt; and as a matter of fact the farmer has been fined—very inadequately—ten pounds. But what it is necessary to point out and insist upon is, that in all these instances, the public must be their own protectors. Paterfamilias should never by any chance take his family to a strange house without making sure of two things: first, that there has been no infectious disease in the house for at least three months before his taking possession; and, secondly, that in the event of there having been infectious disease some time previously, the bedroom of the sick person shall have been thoroughly disinfected. In time we shall probably have an effective system of registration both for lodgings and tenements, whereby risks of all kinds will be very largely diminished. In the meantime let every head of a household be his own detective, and spare no trouble to find out all about the character of a house or lodgings before he entrusts his dearest earthly possessions to its keeping.

---

Enticing to Patients.—An Italian doctor, borrowing a famous line from a great poet and fellow-countryman, has superscribed his door with the words, "Abandon hope all ye who enter here."

